# Supplementary material for: MetaRibo-Seq measures translation in microbiomes
Source: Nat Commun. 2020 Jun 29;11:3268. doi: 10.1038/s41467-020-17081-z (PMC7324362; doi:10.1038/s41467-020-17081-z)
Supplement: Supplementary file 10 — Supplementary Data 7 [file 41467_2020_17081_MOESM10_ESM.zip › File2/Confidence_High_Taxonomy/178758_out.krona.html]

Javascript must be enabled to view this page.

members
magnitude
magnitudeUnassigned
count
unassigned
taxon
rank

178758\_out

31

2
superkingdom
31

1
phylum
976

1
class
200643

171549
order
1

1853231
family
1

1
genus
283168

SRS140492\_contig\_number\_30096

30
phylum
1224

class
1236
30

30
91347
order

family
543
1
30

SRS144219\_contig\_number\_contig-100\_298.299

genus
561
29


SRS012273\_contig\_number\_12424SRS013687\_contig\_number\_contig-100\_671.671SRS014683\_contig\_number\_contig-100\_24262.72491SRS017191\_contig\_number\_contig-100\_1017.111643SRS017521\_contig\_number\_49815SRS018313\_contig\_number\_contig-100\_9699.9699SRS019601\_contig\_number\_contig-100\_23127.102889SRS021153\_contig\_number\_contig-100\_11102.11102SRS046502\_contig\_number\_8616SRS049402\_contig\_number\_13922SRS057478\_contig\_number\_contig-100\_15169.37915SRS063127\_contig\_number\_contig-100\_4802.4803SRS064276\_contig\_number\_22144SRS075947\_contig\_number\_26352SRS077127\_contig\_number\_contig-100\_7197.49707SRS077454\_contig\_number\_13343SRS077646\_contig\_number\_contig-100\_442.443SRS098717\_contig\_number\_4273SRS098881\_contig\_number\_26368SRS1041031\_contig\_number\_contig-100\_35404.68305SRS142781\_contig\_number\_6344SRS143423\_contig\_number\_17145SRS143596\_contig\_number\_contig-100\_327.328SRS144362\_contig\_number\_30884SRS146888\_contig\_number\_15506SRS146996\_contig\_number\_20794SRS147151\_contig\_number\_9736SRS148721\_contig\_number\_contig-100\_14421.165904SRS971275\_contig\_number\_12559
species
562
29
